# The cochlear macrophage in presbycusis: inflammaging, neuroinflammation, and targets for therapeutic modulation

**DOI:** 10.3389/fncel.2026.1930209

**Published:** 2026-10-01

**Authors:** Reef K. Al-Asad, Judith S. Kempfle

**Affiliations:** 1 Department of Otolaryngology, UMass Memorial Medical Center, Worcester, MA, United States; 2 University of Massachusetts Chan Medical School, Worcester, MA, United States; 3 The Eaton-Peabody Laboratories, The Massachusetts Eye and Ear Department of Otolaryngology - Head and Neck Surgery, Boston, MA, United States; 4 Harvard Medical School Department of Otolaryngology - Head and Neck Surgery, Boston, MA, United States

**Keywords:** age-related hearing loss, cochlea, immune response, inflammaging, macrophage, presbycusis

## Abstract

Age-related neurodegenerative diseases and age-related hearing loss are increasing in prevalence globally. Immune cells are novel mediators linking these diseases through shared mechanisms of chronic inflammation and maladaptive repair. In the cochlea, resident macrophages and infiltrating immune cells respond to injury, noise exposure, and aging by adopting activated phenotypes that disrupt synaptic integrity, impair myelination, and promote hair cell and neuronal loss. Similarly, in the central nervous system, microglia, the resident immune cells of the brain undergo age- and disease-associated priming, characterized by exaggerated inflammatory responses, complement activation, and impaired clearance of cellular debris. Emerging evidence suggests that cochlear macrophages and CNS microglia share transcriptional programs and signaling pathways. Importantly, these immune cells also serve as key regulators of repair and regeneration when appropriately modulated, adopting pro-regenerative phenotypes that promote tissue recovery rather than injury. As hearing loss is increasingly recognized as a contributing risk factor for neurodegenerative disease, deepening our understanding of age-related immune responses within the inner ear and the mechanisms governing immune cell modulation may help mitigate this risk while uncovering shared therapeutic targets across these related disease processes.

## 1 Introduction

Age-related hearing loss (ARHL), or presbycusis, is a subset of sensorineural hearing loss, that occurs due to damage to the sensory cells of the inner ear: the hair cells and/or spiral ganglion neurons (SGN). It is increasingly recognized as a multidimensional disease due to many overlapping factors, including age and chronic inflammation, trauma (noise exposure, ototoxic drug use, inner ear disease), and genetics (Eckert et al., 2021). Studies have also suggested the importance of immune dysfunction and chronic inflammation in the pathogenesis of ARHL, similar to key mechanisms found in well-studied neurodegenerative diseases (NDs) (Noble et al., 2022).

## 2 Immune function in the inner ear

### 2.1 Cochlear macrophages

The inner ear has a robust local immune system, which contains cochlear resident macrophages in several locations, including the spiral ligament and spiral ganglion, and in close association with the blood-labyrinth barrier (BLB) (Noble et al., 2022; O’Malley et al., 2016; Liu et al., 2021). Cochlear macrophages (CMs) have many functions, including maintaining homeostasis and BLB integrity, clearing debris of damaged cells or myelin of damaged auditory neurons, and immune surveillance (Liu et al., 2025; Warchol, 2019; Zhang et al., 2025). They express known macrophage/microglia markers, including ionized calcium-binding adapting molecule 1 (IBA1), major histocompatibility complex class type II (MHCII), cluster of differentiation 163 (CD163), and CD68 (Noble et al., 2022; O’Malley et al., 2016). These proteins, expressed in non-diseased human temporal bone samples, emphasize that CMs have both physiologic and pathophysiologic functions (O’Malley et al., 2016). Additionally, a population of CMs, known as perivascular-resident macrophage like melanocytes (PVM/Ms), are directly involved in the BLB (Eckert et al., 2021; Stothert and Kaur, 2021).

CMs are predominantly derived from hematopoietic stem cells and depend on colony-stimulating factor 1 (CSF-1) signaling for differentiation and maintenance: monocytes are stimulated to produce M0 macrophages (Noble et al., 2022). At rest, CMs adopt a ramified form, characterized by elaborate branching processes to continuously survey the local microenvironment (O’Malley et al., 2016; Liu et al., 2021). Upon activation, they transition to an ameboid morphology, retracting their processes and acquiring phagocytic capacity (Figure 1A) (Liu et al., 2021; Liu et al., 2025). They exist along a functional continuum between two canonical states: M1 “classically activated” macrophages, which drive pro-inflammatory responses, and M2 “alternatively activated” macrophages, which promote tissue homeostasis and resolution of inflammation (Guan et al., 2025; Chen et al., 2023).

**Figure 1:**
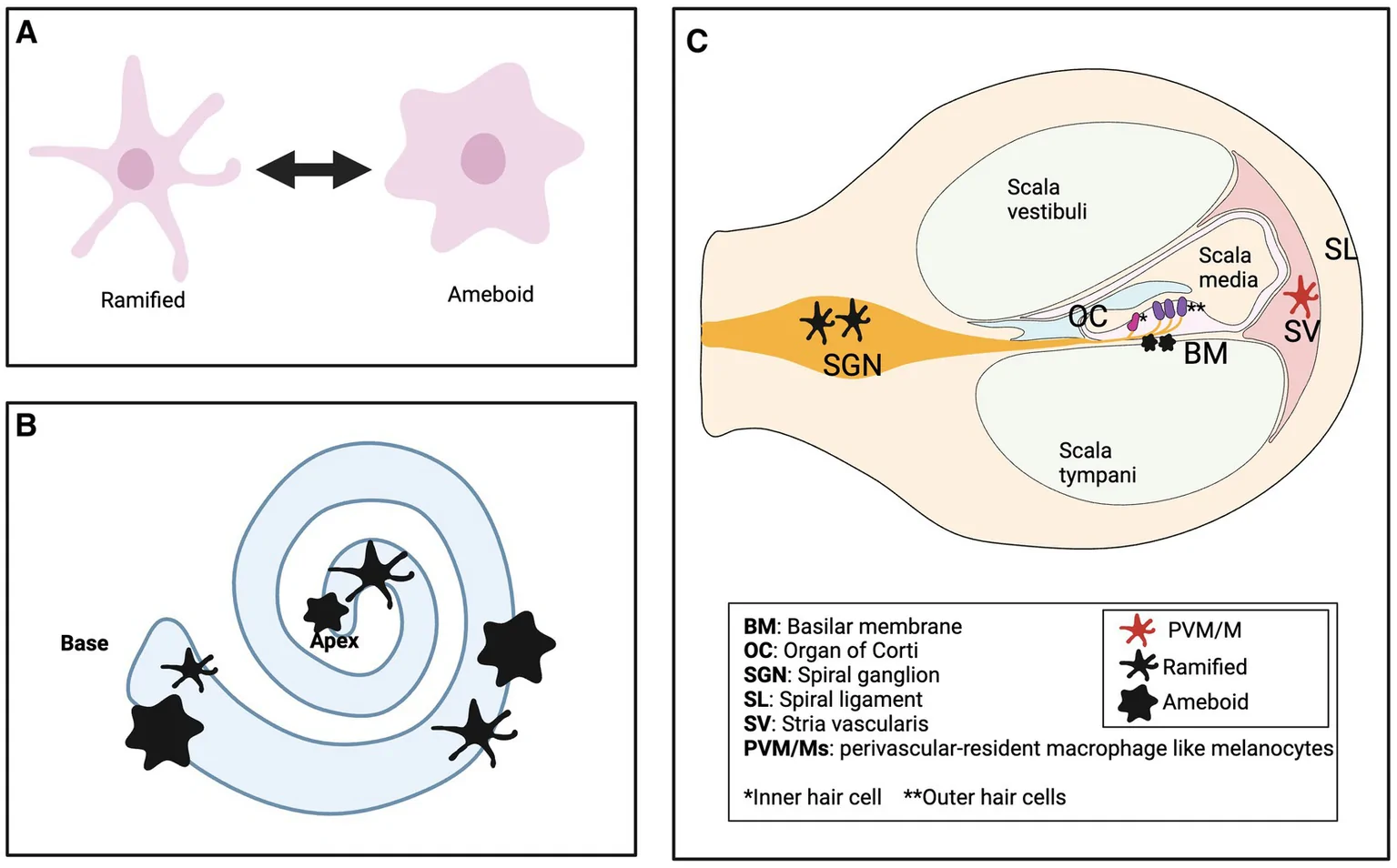
Cochlear macrophages. **(A)** macrophage polarization: macrophages exist between ramified (inactivated) branching morphology and ameboid (activated) morphology. **(B)** Macrophages along the basilar membrane: ramified macrophages are found at the apex, while ameboid macrophages are found at the base. **(C)** Macrophages are located throughout the cochlea in different morphology. Created in BioRender. Kempfle, J. (2026) https://BioRender.com/8am7koc

This morphological landscape is not uniform across the cochlea (Figures 1B,C). In the spiral ganglion, macrophages resemble microglia, extending long processes that contact SGNs and surrounding glia (Warchol, 2019; Zhang et al., 2025). Along the basilar membrane, apical macrophages tend to retain a more ramified, quiescent appearance, while basal CMs adopt an ameboid, activated phenotype (Guan et al., 2025). In mice, this differentiation occurs postnatally (Chen et al., 2023). This progression is also more prominent with age: a pattern that may reflect the base-to-apex progression of age-related cochlear degeneration (O’Malley et al., 2016; Guan et al., 2025; Noble et al., 2019). In the postnatal mouse, macrophages underwent apoptosis in the Organ of Corti (OC) as the cochlea developed further (Chen et al., 2023). In the OC, supporting cells are able to take on immune cell functions. Interestingly, however, in the OC, activated CMs were observed during chronic phases of cochlear damage and in the aging cochlea, more commonly in the basal turn (Lang et al., 2016). At rest, PVM/Ms. are highly branched and located along the stria vascularis but become ameboid and increase BLB permeability once activated (Noble et al., 2019).

Macrophages are important for cochlear development and undergo dynamic changes throughout the lifetime (Balakrishnan et al., 2020). During embryonic stages, they are widely distributed throughout the cochlea and thought to originate from the yolk sac or the fetal liver and can be classified as CSF-1 dependent or independent, respectively (Balakrishnan et al., 2020). Cochlear macrophages subsequently contract in number and become limited to certain locations within the cochlea, wherein new macrophages become recruited from circulating monocytes, derived from the bone marrow (Balakrishnan et al., 2020). In a mouse model of CSF-1 receptor knockout, only macrophages in the SGN and spiral ligament were depleted (Balakrishnan et al., 2020). PVM/Ms. in the stria vascularis are thought to be derived from infiltrating macrophages in murine and primate studies (Montigny and Kempfle, 2025). During embryonic stages, macrophages had temporal and spatial association with key events of cochlear development, including differentiation of sensory hair cells and pruning of SGNs (Montigny and Kempfle, 2025). Under pathological conditions, monocyte-derived macrophages can be recruited from systemic circulation, which may amplify or modulate the intracochlear inflammatory milieu (Lang et al., 2016).

In the mature cochlea, macrophages support maintenance and survival of SGNs. The neuroprotective significance of this function was demonstrated by the consequences of selective macrophage depletion: administration of PLX5622, a CSF-1 receptor inhibitor, led to significantly greater SGN degeneration both in mice undergoing cochlear implantation and in the unoperated control ears, compared to animals with intact macrophage populations (Li et al., 2026). These findings underscore that CMs are active neuroprotective agents, tempering the inflammatory response and sustaining neuronal integrity under both physiological and stressed conditions.

The interplay between glia and immune cells in the cochlea is fundamental for the development of the inner ear and its sustained function throughout life (Noble et al., 2019). Schwann cells represent the predominant glial population, serving as the architects of auditory neuron myelination. Additionally, following nerve injury, Schwann cells adopt a dynamic regenerative state characterized by re-entry into proliferation, re-expression of developmental transcription factors, and active participation in the clearance of myelin debris (Balakrishnan et al., 2020; Montigny and Kempfle, 2025).

Macrophages are essential to this repair process. They are among the first responders to peripheral nerve injury, clearing cellular debris and shaping the local inflammatory milieu through a context-dependent repertoire of secreted signals. Their capacity to release both pro-inflammatory mediators, such as Tumor necrosis factor (TNF)-α, interleukin (IL)-1β, and IL-6, and anti-inflammatory or neurotrophic factors such as IL-10 and Insulin-like Growth Factor (IGF)-1, positions them as pivotal key regulators of the regenerative balance (Zhang et al., 2025). Through these signals, macrophages directly influence Schwann cell proliferation and regeneration and are therefore central to the restoration of nerve integrity following injury (Zhang et al., 2025).

### 2.2 Macrophage and immune system regulation in the aging cochlea

There is a growing consensus that the pathophysiology of ARHL develops from low-grade chronic inflammation known as inflammaging. The cochlear immune landscape is a complex collection of processes, including surveillance by immune cells, protection from systemic circulation by the BLB, and mechanisms that counteract accumulation of reactive oxygen species (ROS) (Li et al., 2026). The proportion of activated, pro-inflammatory macrophages is increased in the aging cochlea (Frye et al., 2017). Aberrant activation of the local immune system and CMs is implicated in this.

#### 2.2.1 Macrophages are increasingly activated with age

In mouse models of presbycusis, activated macrophages were observed in the cochlea prior to detectable hair cell damage and were concentrated near hair cells that subsequently degenerated (Frye et al., 2017). This suggests that macrophage activation may drive, rather than simply accompany, sensory cell loss. Consistent with this, pro-inflammatory macrophages were identified in the cochleae of aging mice (Lang et al., 2023), and RNA sequencing of cochlear tissue in mouse models of ARHL revealed a transcriptionally distinct expansion of pro-inflammatory macrophage populations (Wu et al., 2025).

Analysis of human temporal bone samples from older donors revealed a markedly increased proportion of activated macrophages in the auditory nerve (AN) and the lateral wall - two structures whose integrity is central to auditory function and degeneration is a hallmark of presbycusis (Noble et al., 2022). As in other tissues, macrophages in the cochlea adopt increasingly pro-inflammatory phenotypes with advancing age, perpetuating a chronic inflammatory microenvironment that progressively undermines cochlear homeostasis (Parekh and Kaur, 2023).

#### 2.2.2 Perivascular-resident macrophage-like melanocytes

In the BLB, the number of perivascular-resident macrophage-like melanocytes decreased in aged ears; however, a larger proportion of these remaining cells were activated, suggesting macrophage loss and dysfunction in the BLB may contribute to ARHL (Noble et al., 2022; Neng et al., 2015). Moreover, increased interaction with systemic circulation due to increased BLB permeability allows for increased macrophage activation in the cochlea (Li et al., 2026).

#### 2.2.3 Signaling pathways involved in macrophage activation

Molecular pathways that govern cochlear immune homeostasis include coordinating macrophage recruitment, regulating the intracochlear inflammatory response, and providing neuroprotection in the setting of damage. Signaling pathways implicated in macrophage function and activation may include, but are not limited to: Fractalkine signaling pathway, JAK/STAT (Janus Kinases/ signal transducer and activator of transcription) signaling pathway, Phosphoinositide-3-kinase/protein kinase B (PI3K/Akt) pathway, MAPK signaling pathway, mechanistic target of rapamycin (mTOR) signaling pathway, NOD-, LRR- and pyrin domain-containing protein 3 (NLRP3) inflammasome pathway and Nuclear Factor kappa B (NF-kB) pathway.

Fractalkine (CX3CL1) is a chemokine constitutively expressed on SGNs and hair cells that binds exclusively to the CX3CR1 receptor on immune cells, including CMs. The pathway is upregulated during inflammation and has been implicated in both neurodegenerative disease and ARHL (Stothert and Kaur, 2021). Its functions include immune cell recruitment and adhesion, synaptic pruning during central nervous system (CNS) development, neuron–glia communication, and the neuroprotection of SGNs through suppression of pro-inflammatory macrophage activity and promotion of anti-inflammatory polarization (Stothert and Kaur, 2021). The consequences of disrupting this pathway are significant. CX3CR1 knockout mice exhibit increased SGN loss accompanied by elevated IL-1β expression, similar to observations in the CNS, and points toward neuroinflammation as the mechanistic basis of SGN vulnerability (Kaur et al., 2018). Relative to wildtype controls, mouse models with absent or reduced fractalkine signaling demonstrate increased hair cell and SGN death, impaired spontaneous ribbon synapse recovery following noise exposure, diminished macrophage recruitment after selective hair cell damage, and reduced SGN survival (Kaur et al., 2019; Kaur et al., 2015). Under homeostatic conditions, however, macrophage numbers appear unaffected by disruption of the pathway, suggesting that its protective functions are most consequential in the context of injury or stress (Stothert and Kaur, 2021). CX3CR1 knock out mice that underwent noise exposure demonstrated significantly increased neutrophil infiltration that was associated with worse hearing outcomes and hair cell loss as compared to controls, further suggesting a role for fractalkine signaling in regulation of the immune microenvironment (Frye et al., 2017).

The JAK/STAT signaling pathway plays an important role in macrophage polarization. Specifically, STAT3 has been shown to promote M2 polarization in multiple tissues (Lang et al., 2023). The PI3K/AKT signaling pathway is involved in both M1 and M2 phenotypes. Downregulation of the AKT1 isoform favors M1 polarization, while downregulation of the AKT2 isoform promotes the anti-inflammatory M2 phenotype and a dampened inflammatory response (Wu et al., 2025). The kinase mTOR, inhibited by rapamycin, has been implicated in age-related neuroinflammation and is increasingly studied in the context of inner ear disease. The mTOR pathway, divided into mTOR complex 1 and complex 2 (mTORC1, C2 respectively), plays many roles, including regulation of autophagy, synthesis of cellular components essential for cell survival and cell growth and survival (Parekh and Kaur, 2023). Newer evidence suggests overactivation of the mTOR pathway, secondary to dysregulated upstream activators, may lead to SNHL. MTOR signaling falls downstream to PIK3/AKT signaling and may play a role in M2 activation as well (Parekh and Kaur, 2023; Neng et al., 2015). Mitogen activated protein kinase (MAPK) signaling is also involved in macrophage activation and polarization, typically activated downstream to toll-like receptor (TLR) signaling (Stothert and Kaur, 2021).

The broader cochlear inflammatory environment in aging is further destabilized by a failure of oxidative stress regulation. Mechanisms that clear ROS are progressively weakened in the aging cochlea, lowering the threshold for activation of downstream inflammatory cascades. Chief among these is the NLRP3 inflammasome — a multi-protein complex whose overactivation is implicated in the pathogenesis of neurodegenerative disorders and ARHL (Parekh and Kaur, 2023). NLRP3 activation follows a two-step process: an initial priming signal, followed by activation triggered by pathogen- or damage-associated molecular patterns. This leads to caspase-1 activation, release of pro-inflammatory cytokines IL-1β and IL-18, and pyroptotic cell lysis, flooding the local microenvironment with inflammatory mediators. Priming can be driven by upstream inflammatory pathways including (TLR)9/NF-κB signaling, which elevates expression of IL-6 and TNF-α (Parekh and Kaur, 2023). IL-1β, in turn, is known to recruit microglia in the CNS, establishing a self-reinforcing cycle of neuroinflammation. Dysregulation of the complement cascade represents an additional amplifying mechanism, contributing to cochlear tissue damage through pathways shared with broader neurodegenerative disease processes (Parekh and Kaur, 2023).

### 2.3 Interactions with neurons and glial cells

CMs also interact with the myelinating glial cells of the AN. During development, macrophages actively regulate glial cell populations; in the aging AN, their numbers increase alongside a parallel rise in macrophage-glia interactions – a pattern that implicates them in the progressive structural deterioration we see in presbycusis (Brown et al., 2017). Macrophages and Schwann cells engage directly within the cochlea and AN, and macrophages influence glial function, including the regulation of myelination, through these contacts (Noble et al., 2019; Brown et al., 2017). Consistent with this, elevated numbers of activated macrophages have been documented in the cochleae and ANs of older human donors (Noble et al., 2019). When macrophages adopt a pro-inflammatory phenotype, this relationship becomes deleterious: inflammatory macrophages may directly impair Schwann cell function, compromising myelination of auditory neurons and thereby contributing to AN degeneration (Zhang et al., 2025).

An interesting site of pathology is the glial transition zone along the AN, where responsibility for axonal myelination shifts from Schwann cells of the peripheral nervous system (PNS) to oligodendrocytes in the CNS (Payne et al., 2026). In aged mice and human temporal bone specimens, macrophages/microglia are increased at the transition zone and are found to contain internalized myelin: a hallmark of impaired myelin turnover that is well-recognized in neurodegenerative disease and reflects phagocytic dysfunction and pro-inflammatory polarization (Payne et al., 2026). Evidence of disrupted myelination and dysregulated myelin turnover extends throughout the AN (Payne et al., 2026). These observations point toward macrophage phagocytic function as a therapeutic target in ARHL.

Strategies aimed at rebalancing macrophage polarization, whether by attenuating the M1 pro-inflammatory phenotype, enhancing M2 anti-inflammatory activity, or both, may preserve Schwann cell integrity and harness the regenerative capacity of glial cells, offering a pathway to slow or prevent the progression of presbycusis.

## 3 Modulation of the immune response

### 3.1 Inhibition of pro-inflammatory pathways

Downstream effectors of the complement system converge on macrophage activation. Evidence from clinical trials for age-related macular degeneration, a disease that shares notable pathophysiological parallels with presbycusis, showed promising results using complement inhibitors (Qin et al., 2021). Increased C1q deposition and microglial activation have been observed in the cochlear nucleus of aged mice, identifying an additional potential locus for intervention in ARHL (Seicol et al., 2022). Complement factor B, a key activating protein of the alternative pathway, has also emerged as unexpectedly important for AN integrity: factor B-deficient mice exhibit progressive hearing loss, SGN loss, and myelin sheath damage despite a structurally intact organ of Corti and normal hair cells — implicating this pathway in glial cell function, myelination, and neural crest development independently of sensory cell pathology (Brown et al., 2023). Further study on the complement system and macrophage-specific changes may uncover new targets as well.

The NLPR3 inflammasome signaling pathway similarly contains many potential targets. Galectin-3, expressed on microglia and macrophages, has emerged as a molecule of interest through its putative link to lysosomal destabilization – a feature of cellular aging that contributes to NLRP3 activation (Noble et al., 2022; Li et al., 2025). Galectin-3 expression rises with age and is associated with neurodegenerative pathology more broadly (Noble et al., 2022; Wang et al., 2015; Yan et al., 2016). Considering the role of Galectin-3 in other diseases, it may serve as a possible target for modulating inflammation in the cochlea. Galectin-3 has also been implicated in age-related hearing outcomes and hair cell damage (Kaur et al., 2018). Interestingly, in a knockout mouse model of Galectin-3, female mice had significantly accelerated ARHL as compared to the male mice or controls (Kaur et al., 2018).

Further upstream, both the TLR4 and TLR9 pathways can independently prime the NLRP3 inflammasome, implicating NF-κB and its downstream effectors — including IL-1 and TNF-α — as additional points of intervention. IL-1β, a principal downstream product of NLRP3 inflammasome activation, plays a well-established role in hearing loss pathogenesis (Kaur et al., 2019). The therapeutic relevance of these cytokines has been demonstrated experimentally: concurrent inhibition of IL-1 and TNF-α using anakinra and etanercept, respectively, protected hearing thresholds in noise-exposed mice (Arpornchayanon et al., 2013; Yaşar et al., 2025). Blockade of IL-6 signaling in noise-exposed mice similarly reduced macrophage activation and improved SGN survival (Wakabayashi et al., 2010). Administration of minocycline, following neomycin-induced cochlear injury in mice was shown to partially preserve hair cells by attenuating macrophage activation (Kaur et al., 2015). Taken together, these findings suggest that modulation of inflammatory signaling, the complement cascade, the NLRP3 inflammasome, or their shared downstream mediators, is a possible strategy for limiting cochlear damage that underlies ARHL.

### 3.2 Upregulation of anti-inflammatory and pro-repair pathways

Given its dual role in inflammatory regulation and neuroprotection across the CNS and PNS, the fractalkine signaling pathway represents a promising therapeutic target for hearing loss and NDs.

Evidence from preclinical models is encouraging: exogenous fractalkine overexpression in a mouse model of tauopathy rescued cognitive function (Finneran et al., 2019), and in animal models of Parkinson’s disease, exogenous overexpression conferred neuroprotection through enhanced neuronal survival, improved motor function, and attenuation of cytokine release (Pabon et al., 2011; Morganti et al., 2012).

Within the cochlea, the pathway’s therapeutic potential has been demonstrated. Local administration of soluble fractalkine to noise-exposed mice restored synaptic integrity and hearing thresholds – but only in the presence of macrophages. Macrophage depletion following noise exposure exacerbated synaptic and neuronal loss and elevated ABR thresholds. Reintroduction of macrophages to the cochlea led to recovery of these parameters, underscoring that macrophages are not only modulators of inflammation but active mediators of functional synaptic regeneration (Manickam et al., 2023). Fractalkine signaling may serve a dual therapeutic opportunity: its anti-inflammatory properties can protect cochlear structures before damage accrues, and its restorative potential may rescue following injury. Its activity across both the CNS and PNS broadens its potential relevance, positioning it as a candidate not only for presbycusis but for other neuroinflammatory and neurodegenerative conditions.

## 4 Repair and regeneration

The regenerative potential of CMs extends beyond inflammatory modulation into active tissue repair. In mouse models of auditory neuropathy, increased infiltration of hematopoietic stem cell-derived macrophages into the AN was observed and is thought to contribute meaningfully to repair and regeneration through clearance of damaged cellular debris and promotion of glial cell proliferation (Lang et al., 2016). This regenerative capacity may be improved through mesenchymal stem cell (MSC)-based approaches: MSCs have been shown to suppress pro-inflammatory microglial activation while at the same time enhancing neuroprotective, anti-inflammatory microglial states, in part through activation of the fractalkine signaling pathway (Giunti et al., 2012). MSCs have demonstrated the ability to push macrophage differentiation toward the M2 anti-inflammatory phenotype across multiple organ systems, including pulmonary and endocrine contexts, suggesting a broadly conserved mechanism (Zhang et al., 2021).

A conceptually distinct but complementary strategy is the replacement of aged, dysfunctional macrophages with newly generated, immunologically competent cells. In a mouse model of intracerebral hemorrhage, pharmacological elimination of microglia using a CSF-1 receptor inhibitor was followed by rapid repopulation upon cessation of treatment; the newly generated cells exhibited markedly reduced expression of pro-inflammatory cytokines and activation markers (Li et al., 2022). An analogous dynamic has been observed in the cochlea: elimination of resident macrophages resulted in sustained synaptic and neuronal damage and persistent hearing loss following noise exposure, relative to untreated controls (Manickam et al., 2023). Upon treatment cessation and macrophage repopulation, previously depleted mice recovered hearing thresholds, restored ribbon synapses, and preserved SGNs (Manickam et al., 2023). This is evidence that the incoming macrophage population recapitulated both the protective and reparative functions of its predecessors, and that these functions are essential for normal cochlear function.

Following neuronal damage, cochlear glial cells expand and can acquire neural progenitor characteristics, offering a potential source of neuronal replacement (Kempfle et al., 2020; Lang et al., 2015). Concurrently, macrophages polarize toward a neuroprotective phenotype that is critical for SGN survival and maintenance, suggesting that the immune and regenerative responses are not parallel but interdependent processes (Kaur et al., 2015). Targeted modulation of macrophages toward anti-inflammatory and neuroprotective states may therefore amplify the efficacy of future cell replacement and regenerative therapies in ARHL.

## 5 Discussion

ARHL is increasingly understood as an actively driven pathological process in which immune dysregulation plays a central and potentially initiating role. This review examined the CM, who’s chronically activated, pro-inflammatory state contributes to the progressive degradation of sensory cells, auditory neurons and glial integrity, as a central mediator of presbycusis. Three therapeutic strategies emerge from this literature: suppression of pro-inflammatory signaling cascades, upregulation of anti-inflammatory and neuroprotective pathways, and macrophage replacement. Because CMs share biological overlap with CNS microglia, insights from ND research offer a valuable first roadmap for the cochlea.

An important caveat is that the immune response to acute cochlear injury is considerably better characterized than that underlying ARHL, and meaningful differences exist between the two. In acute injury, infiltrating monocyte-derived macrophages are the predominant drivers of the inflammatory response; in presbycusis, chronic dysregulation of resident tissue macrophages appears to be the primary pathological cause (Frye et al., 2017). Even subclinical hearing loss may leave an immunological footprint: CMs exposed to low-level noise leading to temporary threshold shifts exhibited activation and reduced phagocytic capacity (Frye et al., 2017). This observation points to macrophage priming, whereby repeated or cumulative insults condition the immune system toward an exaggerated pro-inflammatory response to subsequent stressors (Hough et al., 2022). The cumulative burden of such events over decades may be a significant yet underestimated contributor to the inflammaging trajectory of the aging cochlea. Despite the distinctions between acute and chronic injury paradigms, the two are likely mechanistically related, and therapeutic insights derived from acute models may be relevant for the development of ARHL interventions.

An important principle derived from the literature is that macrophages must be modulated, not eliminated. Complete macrophage depletion appears to worsen outcomes: SGN degeneration accelerates, synaptic repair fails, and hearing loss deepens (Rahman et al., 2025). Macrophage reintroduction following depletion rescues both neuronal survival and hearing thresholds, demonstrating that a healthy macrophage population retains reparative capacity even after a period of absence (Manickam et al., 2023). This evidence firmly argues against broad immunosuppressive strategies and in favor of dampening pathological M1 activity while preserving or enhancing the M2 neuroprotective and pro-repair functions that are essential to cochlear homeostasis. The fractalkine/CX3CR1 axis, the NLRP3 inflammasome pathway and the complement cascade demonstrate promising targets for immune modulation in the cochlea.

Timing of intervention is likely as important as the choice of a target. Macrophage activation and shifts toward pro-inflammatory states have been documented in both animal models and human temporal bone specimens prior to significant hair cell or SGN loss (Seicol et al., 2022), suggesting that the immunological changes that drive structural degeneration precede, and may be necessary for, that degeneration to occur (Noble et al., 2022; Frye et al., 2017). By the time presbycusis is clinically apparent, the inflammatory environment may already be firmly established and the window for meaningful neuroprotection shortened or closed. This argues strongly for preventative or disease-modifying approaches before symptomatic hearing decline. A significant unmet need is audiological and biomarker tools capable of detecting this preclinical immune dysfunction, which would be essential for early intervention strategies.

The broader implications of cochlear immune modulation extend beyond hearing. ARHL has been independently associated with cognitive decline and increased risk of dementia, though the direction and mechanism of this relationship remain incompletely understood. Given that many of the inflammatory cascades implicated in CM dysregulation are shared with Alzheimer’s disease, Parkinson’s disease, and related disorders, therapeutic strategies developed for the cochlea may have direct translational relevance to the brain and vice versa.

In summary, the cochlear macrophage stands at the intersection of protection and pathology in ARHL. A more complete understanding of the signals involved in this balance — and the therapeutic tools to redirect it — offers promise not only for preserving hearing, but for uncovering mechanisms of immune-driven neurodegeneration.

## Glossary

AHRL: Age-related hearing loss
AN: Auditory nerve
BLB: Blood-labyrinth barrier
CD68: Cluster of differentiation 68
CD163: Cluster of differentiation 163
CNS: Central nervous system
CM: Cochlear Macrophages
CSF-1: Colony-stimulating factor 1
IBA1: Ionized calcium binding adapting molecule 1
IL: Interleukin
JAK: Janus kinase
MSC: Mesenchymal stem cell
MTOR: Mechanistic target of rapamycin
ND: Neurodegenerative disease
NLRP3: NOD, LRR- and pyrin domain-containing protein 3
NF-kB: Nuclear factor kappa B
PI3K: Phosphoinositide-3-kinase
PNS: Peripheral nervous system
PVM/Ms: perivascular macrophage-like melanocytes
OC: Organ of Corti
ROS: Reactive oxygen species
SGN: spiral ganglion neurons
STAT: signal transducer and activator of transcription
TLR: Toll-like receptor
TNF: Tumor necrosis factor
